# Effect of Gold Nanoparticles on the Conformation of Bovine Serum Albumin: Insights from CD Spectroscopic Analysis and Molecular Dynamics Simulations

**DOI:** 10.1021/acsomega.4c06409

**Published:** 2024-12-03

**Authors:** Samal Kaumbekova, Naoya Sakaguchi, Dhawal Shah, Masakazu Umezawa

**Affiliations:** † Department of Medical and Robotic Engineering Design, Faculty of Advanced Engineering, Tokyo University of Science, 6-3-1 Niijuku, Katsushika, Tokyo 125-8585, Japan; ‡ Chemical and Materials Engineering, School of Engineering and Digital Sciences, Nazarbayev University, Kabanbay Batyr 53, Astana 010000, Kazakhstan; § Department of Materials Science and Technology, Graduate School of Advanced Engineering, Tokyo University of Science, 6-3-1 Niijuku, Katsushika, Tokyo 125-8585, Japan

## Abstract

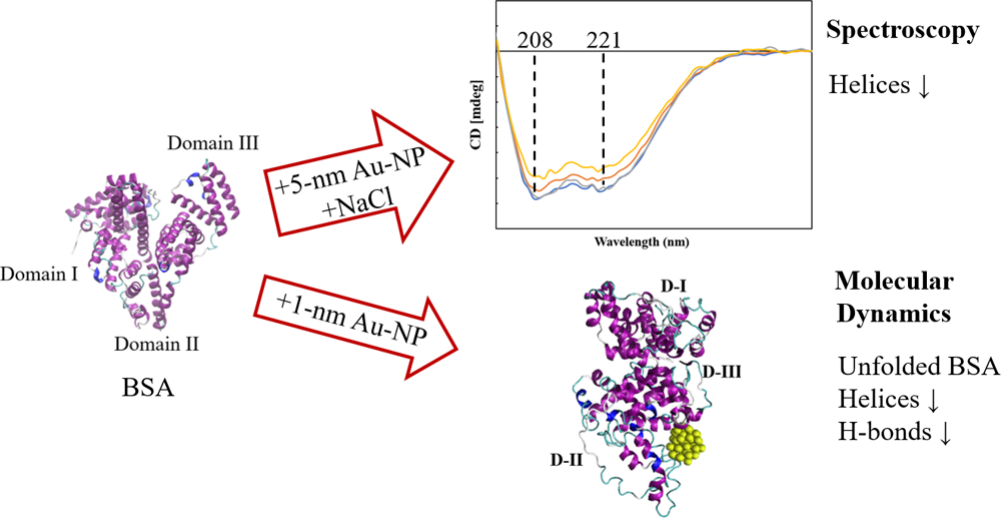

With the development of nanotechnology, there is growing interest in using nanoparticles (NPs) for biomedical applications, such as diagnostics, drug delivery, imaging, and nanomedicine. The protein’s structural stability plays a pivotal role in its functionality, and any alteration in this structure can have significant implications, including disease progression. Herein, we performed a combined experimental and computational study of the effect of gold NPs with a diameter of 5 nm (5 nm Au-NPs) on the structural stability of bovine serum albumin (BSA) protein in the absence and presence of NaCl salt. Circular dichroism spectroscopy showed a loss in the secondary structure of BSA due to the synergistic effect of Au-NPs and NaCl, and Thioflavin T fluorescence assays showed suppressed β-sheet formation in the presence of Au-NPs in PBS, emphasizing the intricate interplay between NPs and physiological conditions. Additionally, molecular dynamics (MD) simulations revealed that 5 nm Au-NP induced changes in the secondary structure of the BSA monomer in the presence of NaCl, highlighting the initial binding mechanism between BSA and Au-NP. Furthermore, MD simulations explored the effect of smaller Au-NP (3 nm) and nanocluster (Au-NC with the size of 1 nm) on the binding sites of the BSA monomer. Although the formation of stable BSA-Au conjugates was revealed in the presence of NPs of different sizes, no specific protein binding sites were observed. Moreover, due to its small size, 1 nm Au-NC decreased helical content and hydrogen bonds in the BSA monomer, promoting protein unfolding more significantly. In summary, this combined experimental and computational study provides comprehensive insights into the interactions among Au nanosized substances, BSA, and physiological conditions that are essential for developing tailored nanomaterials with enhanced biocompatibility and efficacy.

## 1 Introduction

The stability of the protein’s native folded structure and the secondary structure of a protein are vital for its function and activity. The protein unfolding and changes in the secondary structure of the enzymatic or therapeutic protein may lead to the progression of various diseases, such as spongiform encephalopathies or Alzheimer’s Diseases.^1,2^ Among the different factors that might affect the protein structure, the effect of nanoparticles (NPs) is important from the perspective of the toxicity of NPs and the design of nanomaterials with biomedical applications, as was stated in early studies by Lynch et al.^3^ Recent studies have shown that the size, shape, and curvature of NPs are important for their interactions with proteins, intermolecular binding, and the formation of NP-protein complexes, called protein corona (PCs).^4^ For example, a large NP size, associated with a planar surface of NP, resulted in a higher contact surface and stronger interactions with the protein.^5^ In addition, a decrease in the helical content was observed in the chicken egg lysozyme structure in the presence of NPs with a size of 100 nm.^6^

Among the different NPs, the interactions between gold nanoparticles (Au-NPs) and biomolecules are of great interest due to possible applications of Au-NPs in therapeutics, sensing, imaging, drug delivery, and biotechnology.^7^ In addition, gold nanoclusters coated with peptides have been suggested as bioactive therapeutic agents.^8^ Investigation of the formation of PC on gold nanorods revealed the binding structure with the bovine serum albumin (BSA) corona.^9^ Restrictions in the protein structural dynamics due to the binding of BSA to gold nanoclusters (AuNCs) were observed under alkaline conditions, followed by the formation of giant superstructures from the intermolecular aggregation.^10^ In addition, a partial unfolding of the BSA helical structures and decreased β-sheet content was observed in the presence of AuNCs, associated with an alkaline environment used in the synthesis of BSA-AuNCs.^10^ A recent study also showed the altered formation of PC due to the functionalization of Au-NPs.^11^ While investigating the size effect of Au-NPs on the interactions with BSA, varying the NP size from 3.5 to 150 nm, the kinetics of the PC formation was faster in the presence of NPs of a smaller size.^12^

Upon the formation of PC, the binding of Au-NP might affect the stability of the secondary structure of the protein. For example, circular dichroism (CD) analysis of BSA interacting with citrate-stabilized Au-NP (with a size of 20 ± 4 nm) revealed the loss of α-helical structure, as the number of NPs increased from 2.8 × 10^7^ to 5.31 × 10^11^ NP mL^–1^.^13^ Similarly, Shi et al.^14^ observed a high affinity between BSA and Au-NPs owing to the electrostatic interactions between charged Au-NP and surface lysine residues or hydrophobic interactions, resulting in changes in the secondary structure of the protein. The importance of protein concentration and pH conditions in the effect of gold surface on the secondary structure and properties of BSA has been elucidated.^15^ For example, a spectroscopic analysis of the BSA conformation in the albumin-gold-NP bioconjugates was studied at pH 3.8, 7.0, and 9.0, revealing a decrease in helical structures in the bioconjugates with larger changes at higher pH.^16^ In addition, the effect of Au-NPs with different morphologies, such as nanorods, nanospheres, and nanoflowers, on human serum albumin structure was also investigated, suggesting the importance of the size, shape, and structure of metal nanomaterials in their binding ability to proteins.^17^ Moreover, the study on the effect of two types of cationic gemini surfactants at different concentrations showed the unfolding of the BSA structure in AuNP-conjugated BSA due to the binding of surfactants.^18^ In addition, a minor decrease in helical content and the fluorescence quenching of Trp-residues were observed upon bioconjugation with Au-NPs with increasing Au-NP concentrations.^18^

Another factor that may affect the secondary structure of a protein is the possible salting-in and salting-out of proteins in the presence of specific inorganic ions,^19^ depending on their positions in the Hofmeister series. In particular, the weakly hydrated chaotropic ions have a “salting-in” effect, which destabilizes the protein secondary structure and causes protein unfolding.^20^ In contrast, the well-hydrated kosmotropic ions exhibit a “salting-out” effect and promote protein folding.^20^ For example, the α-helical structure is destabilized in the presence of chaotropic ions.^21^ Moreover, our earlier studies demonstrated a synergistic effect of inorganic salts and NPs on the aggregation of amyloid peptides.^22−24^ Although the individual effects of NPs of different sizes^6^ and inorganic ions^25,26^ on the secondary structure of proteins have been studied in the literature, there is still a lack of understanding of the relationship between the binding mechanism of NPs and proteins in the presence of inorganic ions. Moreover, the synergistic effects of NPs and inorganic ions on the secondary structure of the proteins remain unclear.

This study aimed to implement spectroscopic analyses and computational simulations to elucidate the synergistic effect of Au-NPs and the NaCl salt on the BSA conformation, selected as a protein with high stability. In particular, circular dichroism (CD) spectroscopy and the thioflavin T (ThT) fluorescence assay were used to perform experimental analyses. Moreover, considering that the interactions between proteins and NPs have been previously characterized in literature via molecular dynamics (MD) studies,^9^ MD simulations were performed to investigate the stability of the BSA conformation at various sizes of gold nanosized substances (Au-NS) and to elucidate the protein binding sites. Taking into account the limitations of the simulation box size commonly used in MD studies, the largest size of the Au-NP used in the simulations was limited to 5 nm. Consequently, the effect of Au-NPs with comparatively small sizes (3 and 5 nm) and Au-NC with a 1 nm size was explored. Moreover, the impact of specified Au-NPs and Au-NC in the NaCl environment (0.15 M) was studied, considering the physiological conditions.

## 2 Results and Discussion

### 2.1 Circular Dichroism (CD) Spectroscopy

**Figure 1:**
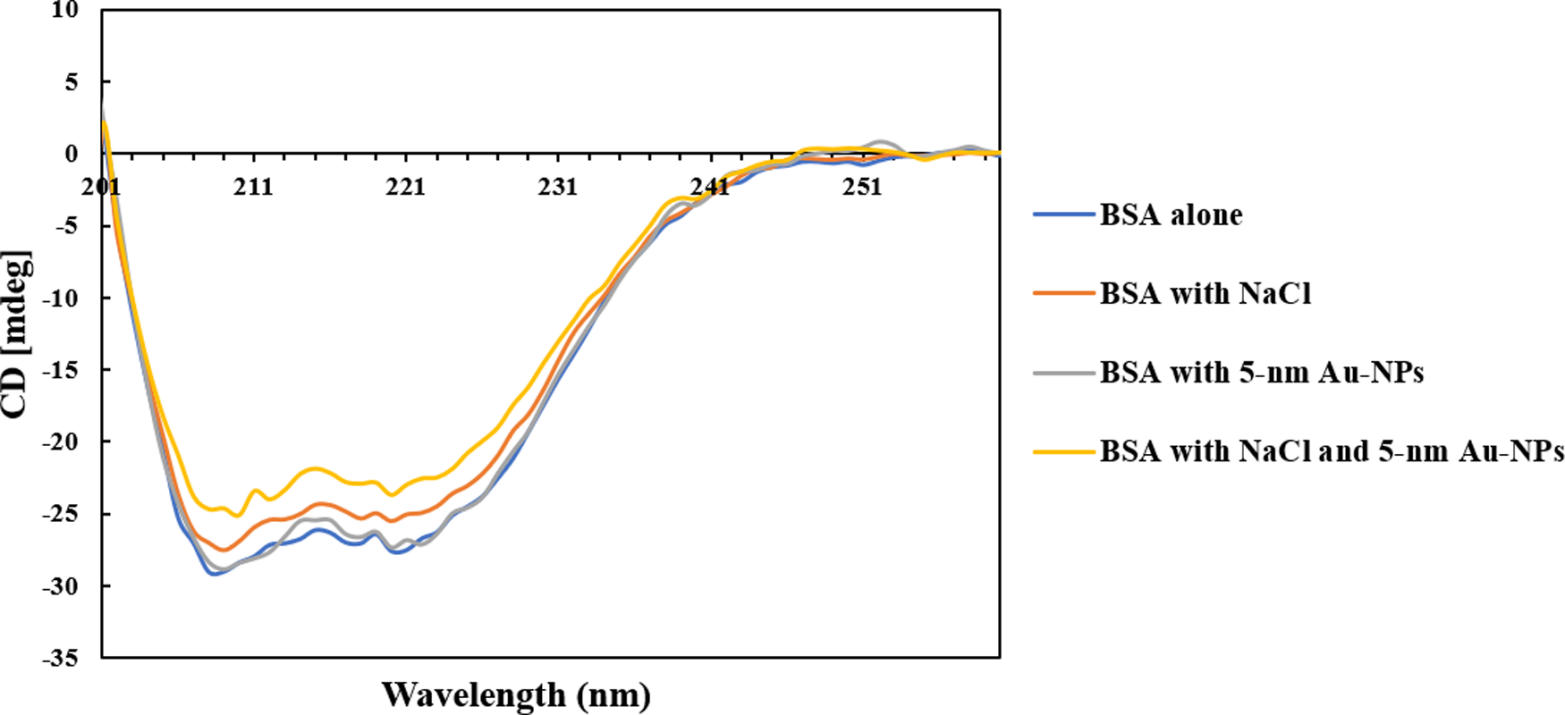
CD spectroscopy of the samples investigated in this study with the enlarged region around 201–260 nm.

CD spectroscopy measurements were performed to investigate the changes in the secondary structure of BSA in the absence and presence of 5 nm Au-NPs and NaCl (Figure 1). According to Figure 1, two specific CD spectroscopy peaks were observed at around 208 and 221 nm for the sample with native BSA, corresponding to the α-helical structure of the protein, consistent with the literature.^10,27^

According to Figure 1, similar characteristic peaks were observed in the sample of BSA with Au-NPs in the absence of NaCl, indicating the stability of the protein structure. While the loss of helical structures in BSA was observed in the presence of citrate-stabilized Au-NPs in previous experimental studies,^13^ considering the difference in the type, size, and charge of the Au-NPs used in our study, the results of our experiments showed no loss of helical structure in the presence of PBS-stabilized Au-NPs with a neutral charge in the absence of NaCl. The results were consistent with the observations from the isothermal titration calorimetry (ITC) of Prozeller et al.,^28^ who showed the weakest interactions between proteins and hydrophilic NPs with a neutral charge, in comparison to the NPs with high surface charge and high hydrophobicity.

In comparison, decreased ellipticity was observed in the samples of BSA in the presence of NaCl, both in the absence and in the presence of Au-NPs, indicating the changes in the secondary structure of BSA (Figure 1). In particular, the most significant changes in the BSA secondary structure were observed in the presence of NaCl and Au-NPs, with the ellipticity loss by 15%, indicating the partial loss of the helical structures. Overall, while 5 nm Au-NPs did not alter the BSA secondary structure in the absence of NaCl, the presence of Au-NPs and NaCl synergistically induced changes in the protein conformation.

### 2.2 Thioflavin T (ThT) Fluorescence Assay

**Figure 2:**
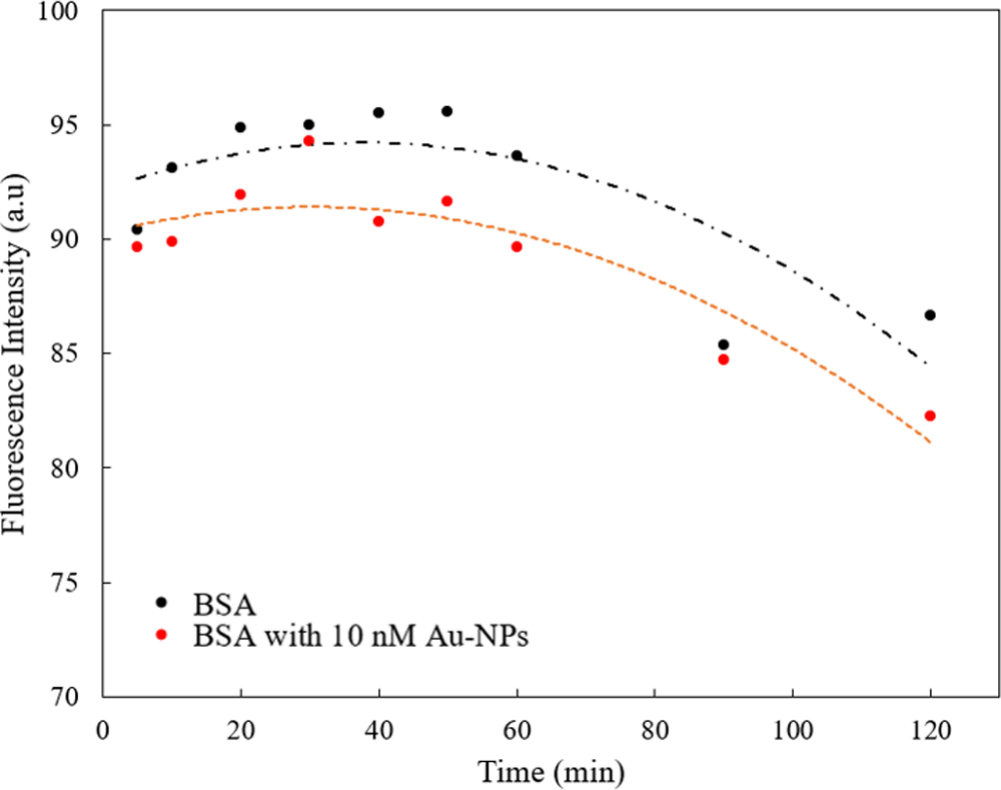
ThT fluorescence measurement results recorded within 120 min from the start of stirring: · BSA with no Au-NPs; · BSA with 10 nM Au-NPs (*d* = 5 nm); -·- polynomial fit for the “BSA with no Au-NPs”; - - - polynomial fit for the “BSA with 10 nM Au-NPs (*d* = 5 nm)”.

Changes in ThT fluorescence intensity were tracked within 120 min of the start of stirring in the samples with BSA in PBS in the absence and presence of 10 nM Au-NPs, as shown in Figure 2. Both samples in the study exhibited an increasing trend for 30 min from the start of stirring. According to Figure 2, for the BSA in the absence of Au-NPs, the ThT intensity reached the maximum value of 96 au within 50 min, followed by a decreasing trend until 120 min (86 au). In contrast, for BSA in the presence of Au-NPs, the ThT intensity reached a maximum value of 94 au within 30 min and then showed a decreasing trend with an outlier at 120 min (82 au). Considering that overall the ThT intensity was higher for the system with no Au-NP, this result suggests that the presence of Au-NPs suppressed the formation of a β-sheet structure in BSA in a phosphate-buffered saline (PBS) environment. The presence of β-sheet content in the BSA structure in our experiments was in line with the experimental results of Kluz et al.,^10^ who observed enhanced ThT emission for native BSA and decreased ThT fluorescence in the presence of AuNCs, associated with the suppressed building-up of a β-sheet conformation in protein-stabilized AuNCs.

### 2.3 MD Simulations: Effect of 5 nm Au-NP on the Structure of the BSA Monomer

At the beginning of the MD study, the selected force field parameters of the BSA structure and water model were validated by simulating the protein monomer in water in the absence of Au-NP. The time evolution of the total solvent accessible surface area (SASA) and radius of gyration (RoG) of the BSA monomer is shown in Figure S1. Our results (in the presence of salt: SASA_40–50 ns_ = 307 ± 7 nm^2^ and RoG_40–50 ns_ = 2.77 ± 0.06 nm, in the absence of salt: SASA_40–50 ns_ = 306 ± 4 nm^2^ and RoG_40–50 ns_ = 2.75 ± 0.03 nm) were in agreement with the reported literature^29,30^ (SASA at approximately 330 nm^2^, RoG at approximately 2.7 nm).

**Figure 3:**
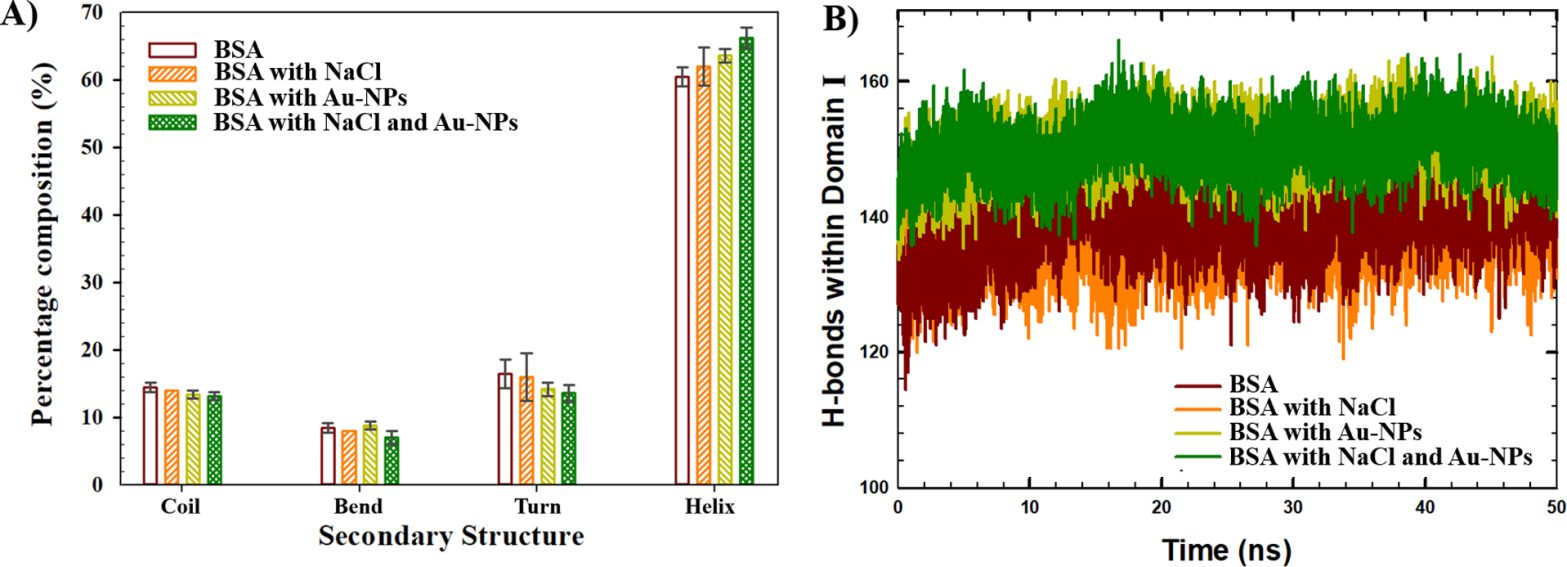
(A) Secondary structure of BSA monomer observed at the last 25 ns of the simulations. (B) Time evolution of H-bonds in Domain I in the absence and presence of Au-NP (*d* = 5 nm), averaged among different runs.

MD simulations were performed to investigate the impact of Au-NP (*d* = 5 nm) on the structure of the BSA in the context of the secondary structure, H-bonds, and SASA analyses (Figure 3). Figure 3A shows the results of the secondary structure analyses averaged over the last 25 ns of the simulations when the systems were equilibrated. The secondary structure of the final protein conformation was compared with that of the initial conformation before the MD simulation (observed at the beginning of the NVT step). The initial percentage composition of the secondary structure of BSA was as follows: 73.8% helices, 13.0% coils, 5.3% bends, and 7.9% turns. As shown in Figure 3A, in the absence of NP, the average percentage amounts of helices observed at the end of the simulations were 62.0 ± 2.8% and 60.5 ± 1.4% in the presence and absence of NaCl, respectively. In comparison, the presence of 5 nm Au-NP resulted in a slightly increased percentage number of helices, such as 66.2 ± 1.5% and 63.6 ± 1.0% in the presence and absence of the salt, respectively. Consequently, the results indicate a partial loss of helical structures within 50 ns of the simulations from the initial BSA conformation. However, since the lowest changes were observed in the presence of NaCl and 5 nm Au-NP, it was suggested that NaCl and 5 nm Au-NP suppressed the loss of the helical structure, indicating the importance of the synergistic effect of Au-NP and NaCl.

Moreover, according to Figure 3A, the compositions of the coils and turns decreased in the presence of Au-NP, both in the absence and in the presence of NaCl, indicating the loss of unstructured regions in the BSA monomer. In particular, in the presence of NaCl, the percentage amounts of coils (14.0 ± 0%) and turns (16.0 ± 3.5%) observed in the absence of Au-NP declined to the values of 13.2 ± 0.6% coils and 13.6 ± 1.2% turns in the presence of NP. In addition, in the absence of salt, the percentage amounts of coils (14.5 ± 0.7%) and turns (16.5 ± 2.1%) observed in the absence of NP declined in the presence of NP to 13.4 ± 0.6% coils and 14.2 ± 1.0% turns.

In addition, the effect of the 5 nm Au-NP on the BSA monomer structure was further explored in terms of the H-bonds associated with protein stability.^31^ In particular, H-bonds were calculated within three different domains (intradomain H-bonds) and between domains (interdomain H-bonds) in the BSA monomer and averaged over different runs within the last 10 ns of the MD simulations. According to the results of the H-bond analyses, although no noticeable difference was observed in the total number of H-bonds (Figure S2), a significant difference was observed in the number of H-bonds within Domain I in the presence of Au-NP. In particular, in the absence of NP, the average number of H-bonds within Domain I was 139 ± 4 and 142 ± 4 in the presence and absence of salt, respectively. With the addition of the Au-NP, the number of H-bonds within Domain I increased up to 151 ± 4 (in the presence of salt) and 151 ± 3 (in the absence of salt), indicating the enhanced stability of the BSA monomer. The time evolution of the H-bonds within Domain I averaged over different runs of the simulated systems is shown in Figure 3B.

Furthermore, SASA analysis was performed to investigate the possible folding or unfolding of the BSA monomer in the systems under the study, which was recognized by the decreased or increased SASA values within the simulation. According to the results, the SASA values of the BSA monomer were approximately 320 nm^2^ at the beginning of the simulations. In the systems without NP, the SASA decreased to 306 ± 4 nm^2^, averaged over the last 10 ns of the simulations. In contrast, in the presence of the 5 nm Au-NP and salt, the SASA was approximately 298 ± 4 nm^2^ at the end of the simulation, indicating a comparatively folded monomeric structure. In summary, according to the results of our MD simulations, an elevated number of H-bonds was associated with high amounts of helices, and a comparatively more folded structure (low SASA values) was observed in the presence of the 5 nm Au-NP and salt. These results were consistent with the literature studies,^32,33^ which correlated the unfolded protein structures with a low amount or lack of the H-bonds, while the secondary structure motifs with a high propensity to form folded structures were usually related to a high number of H-bonds.

Although the β-sheet structures could not be observed in the MD simulations, the results of our MD study showed decreased amounts of the unstructured regions (coils and turns) and suppressed the loss of helices in the presence of the 5 nm Au-NP and NaCl, correlated with a stable folded monomer structure (low SASA and high amounts of intradomain H-bond). In agreement with the Monte Carlo simulations performed by Sharma et al.,^34^ as the secondary structure of the protein might change after the adsorption, the protein prefers to remain folded to avoid the significant loss of entropy in its unfolded state upon adsorption to surfaces. Although previous MD studies investigated the changes in protein conformational entropy, efficient and accurate calculations of protein backbone entropy on an atomistic level require high computational resources.^35^ Considering the size and complexity of our systems and the limitations of our computational resources, calculations of the entropy changes were out of the scope of the present study.

Overall, the MD results showed induced changes in the BSA conformation due to the synergistic effect of Au-NPs and NaCl, consistent with the results of our experiments. Considering that the BSA monomer does not have β-sheets in its native monomeric form, the β-sheets were not observed in the monomeric structure at 50 ns of the MD runs. Our results were consistent with the literature, which showed the predominance of the helices and absence of the β-sheets in the BSA monomer structure in 100 ns of the MD run^29^ and 300 ns of the MD simulations.^36^ Nevertheless, taking into account the limitations in the MD simulation time and box size, the simulations indicated the initial mechanism of the protein-NP interactions and protein corona formation between a BSA monomer and a single Au-NP within 50 ns. In particular, MD simulations showed a comparatively high amount of helices in the presence of Au-NP and NaCl during the initial binding of the BSA monomer to Au-NP. Taking into account that in the MD simulations, one Au-NP was available for the interactions with a single BSA monomer, resulting in the formation of a PC with high helical content. However, the long-term effect in the presence of higher concentrations of BSA and Au-NPs will further induce changes in the protein conformation, decreasing the helical and β-sheet content, as shown in our experiments, highlighting the importance of the protein and NP concentrations. This observation was also reported in the previous experimental studies, which showed the induced loss of the albumin secondary structure, as the concentration of Au-NPs increased.^13,18^

### 2.4 MD Simulations: Size Effect of Au-NS on the Structure of the BSA Monomer

The effect of the Au-NS size on the secondary structure of the BSA monomer was further studied in the absence and presence of 0.15 M NaCl (Table 1). Considering the limitations of the simulation box size, the Au-NP with diameters of 3 and 5 nm, and Au-NC with the size of 1 nm were used for the simulations. The initial distances between the center of mass of the BSA monomer and Au-NS in all of the simulated systems were in the range of 3–5 nm (Table 1). The results for the protein SASA and H-bonds observed in the last 10 ns of the simulated systems are summarized in Tables 1 and 2.

**Table 1:** Initial Distances between BSA and Au-NS, Final Protein SASA, Intraprotein H-bonds, and BSA Secondary Structure Percentage Values Were Averaged among Different Runs of the Simulated Systems

Au-NS	no NP		1 nm Au-NC		3 nm Au-NP		5 nm Au-NP	
NaCl	-	0.15 M	-	0.15 M	-	0.15 M	-	0.15 M
initial distances between BSA and Au-NS (nm)			4.7 ± 1.3	4.1 ± 2.0	3.6 ± 0.6	3.7 ± 1.3	3.6 ± 0.6	3.1 ± 0.9
SASA_final_ (nm^2^)	306 ± 4	307 ± 7	317 ± 12	308 ± 5	307 ± 10	293 ± 8	309 ± 5	298 ± 4
H-bonds_final_	449 ± 12	443 ± 15	423 ± 12	440 ± 12	448 ± 11	454 ± 11	455 ± 11	466 ± 15
coil_final_ (%)	14.5 ± 0.7	14.0 ± 0.0	15.7 ± 0.6	14.6 ± 0.6	15.0 ± 0.0	14.0 ± 0.0	13.4 ± 0.6	13.2 ± 0.6
bend_final_ (%)	8.5 ± 0.7	8.0 ± 0.0	11.3 ± 1.5	8.6 ± 1.2	8.2 ± 1.2	8.3 ± 0.6	8.8 ± 0.6	7.0 ± 1.0
turn_final_ (%)	16.5 ± 2.1	16.0 ± 3.5	18.3 ± 2.3	15.6 ± 1.5	16.2 ± 1.5	15.3 ± 0.6	14.2 ± 1.0	13.6 ± 1.2
helix_final_ (%)	60.5 ± 1.4	62.0 ± 2.8	54.7 ± 2.5	61.2 ± 1.5	60.6 ± 1.2	62.4 ± 1.5	63.6 ± 1.0	66.2 ± 1.5

**Table 2:** Intradomain and Interdomain H-bond Numbers Averaged over the Last 10 ns of the Simulations in Different Runs

Au-NS	no NP		1 nm Au-NC		3 nm Au-NP		5 nm Au-NP	
NaCl	-	0.15 M	-	0.15 M	-	0.15 M	-	0.15 M
Domain I–I	142 ± 4	139 ± 4	136 ± 6	143 ± 5	148 ± 6	145 ± 5	151 ± 3	151 ± 4
Domain II–II	130 ± 6	129 ± 5	121 ± 5	128 ± 5	126 ± 5	131 ± 5	131 ± 6	131 ± 5
Domain III–III	138 ± 6	140 ± 6	129 ± 6	134 ± 5	133 ± 6	136 ± 5	138 ± 6	144 ± 5
Domain I–II	6 ± 1	6 ± 2	4 ± 1	6 ± 1	9 ± 2	9 ± 1	7 ± 2	9 ± 1
Domain I–III	6 ± 1	6 ± 2	7 ± 2	5 ± 1	6 ± 2	7 ± 2	5 ± 2	4 ± 1
Domain II–III	5 ± 1	4 ± 1	7 ± 2	5 ± 1	6 ± 1	6 ± 1	5 ± 1	6 ± 1

According to Tables 1 and 2, among the Au-NPs of different sizes, the highest average number of H-bonds (466 ± 15 total H-bonds and 151 ± 4 intradomain H-bonds in Domain I) was correlated with the highest average helix content (66.2 ± 1.5%) and the low average final SASA (298 ± 4 nm^2^) in the presence of 5 nm NP and NaCl. This observation also revealed a stable and compact folded BSA structure, indicating that 5 nm NP could stabilize the BSA monomer in the presence of salt. Considering the effect of NaCl on the secondary structure of the BSA monomer, Na^+^ and Cl^–^ ions are located on the borderline between the kosmotropic and chaotropic ions in the Hofmeister series, which shows no definite effect on the protein structure.^20^ However, in our study, the presence of both 5 nm Au-NP and NaCl resulted in the enhanced folding of the protein and formation of H-bonds, indicating a distinct synergistic effect of Au-NP and NaCl.

In comparison, in the presence of small-sized 1 nm Au-NC and no salt conditions, the average number of H-bonds observed in the last 10 ns of the simulations was comparatively low, with approximately 423 total H-bonds (Table 1) and 151 intradomain H-bonds in Domain I (Table 2). Moreover, a high number of coils (15.7 ± 0.6%), bends (11.3 ± 1.5%), and turns (18.3 ± 2.3%), and the lowest number of helices (54.7 ± 2.5%) were observed in the system with 1 nm Au-NC in the absence of salt, which also corresponded to the highest average final SASA (317 ± 12 nm^2^). The results indicated that the BSA monomer with an approximate size of 4–7.5 nm^37^ (or higher when unfolded) could lose its stability and unfold due to high interference and possible embedment of the 1 nm Au-NC with a significantly smaller size into the gaps between the flexible protein domains. This observation was consistent with our previous study on the enhanced interactions between small molecules and a protein monomer.^38^ In addition, as was observed from the molecular docking analysis of BSA with Au-NCs of 1.8 nm size by Halder et al.,^18^ Au-NCs could denature helical content with the most stable docking positions near the surface of the protein. Our results were in agreement with Lynch et al.,^3^ who hypothesized that the adsorption of NPs with small size and high curvature might cause the highest destruction to the secondary structure of a protein.

**Figure 4:**
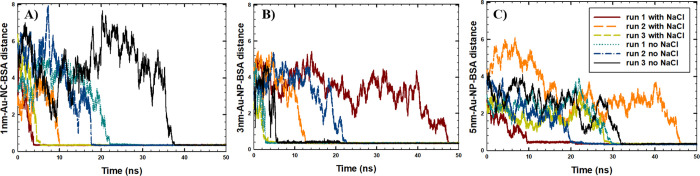
Time evolution of the intermolecular distances (nm) between the center of masses of (A) BSA monomer and 1 nm Au-NC, (C) BSA monomer and 3 nm Au-NP, and (B) BSA monomer and 5 nm Au-NP. Three runs of the simulated systems with NaCl and three runs of the simulated systems with no NaCl are shown separately for each system.

**Figure 5:**
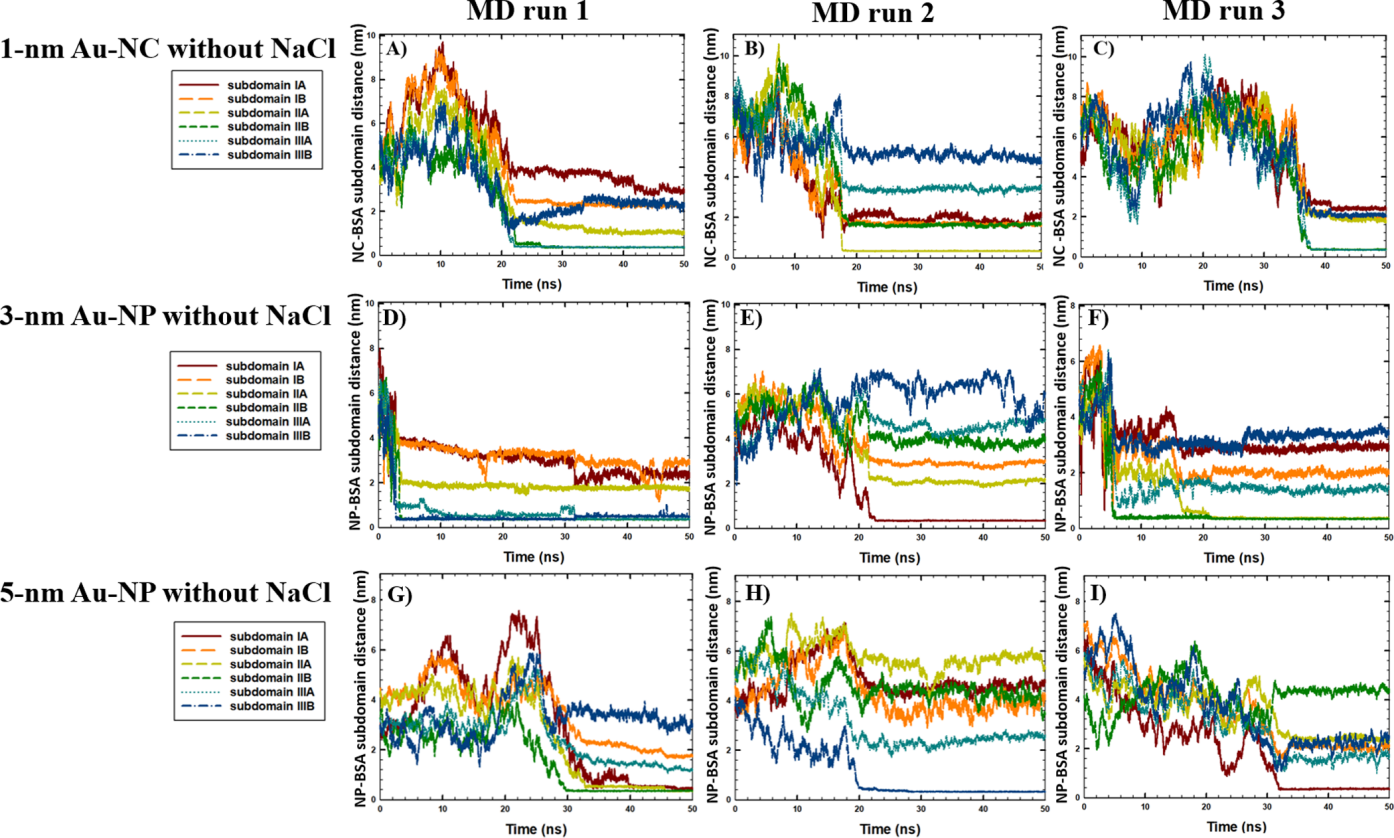
Time-evolution of the distances between the COM of the Au-NS and BSA monomer subdomains in the absence of NaCl in (A) run 1 (1 nm Au-NC), (B) run 2 (1 nm Au-NC), (C) run 3 (1 nm Au-NC), (D) run 1 (3 nm Au-NP), (E) run 2 (3 nm Au-NP), (F) run 3 (3 nm Au-NP), (G) run 1 (5 nm Au-NP), (H) run 2 (5 nm Au-NP), and (I) run 3 (5 nm Au-NP).

Next, to investigate the possible aggregation of BSA and Au-NS of different sizes, the time evolution of the intermolecular distances between Au-NS and BSA was studied (Figure 5). The aggregation time between BSA and Au-NS was estimated based on the criteria of the distance between the center of masses (COM) of the BSA and Au-NS taken as 0.4 nm. As shown in Figure 4, the BSA monomer and Au-NS aggregated within 50 ns in all of the simulated runs, reaching an intermolecular distance between the COM of BSA and Au-NS of 0.4 nm. In addition, the formation of stable BSA-NP conjugates was observed in all runs, considering that the distance between BSA and Au-NS did not change after the aggregation occurred. Furthermore, as shown in Figure 4, the fastest aggregation kinetics was observed between BSA and 1 nm Au-NC in the presence of NaCl (6.4 ns), averaged among the three simulation runs. Our results were consistent with the results of the experimental study of Piella et al.,^12^ who showed faster aggregation kinetics between BSA and Au-NPs of a smaller size (among the NPs with diameters of 3.5–150 nm).

**Figure 6:**
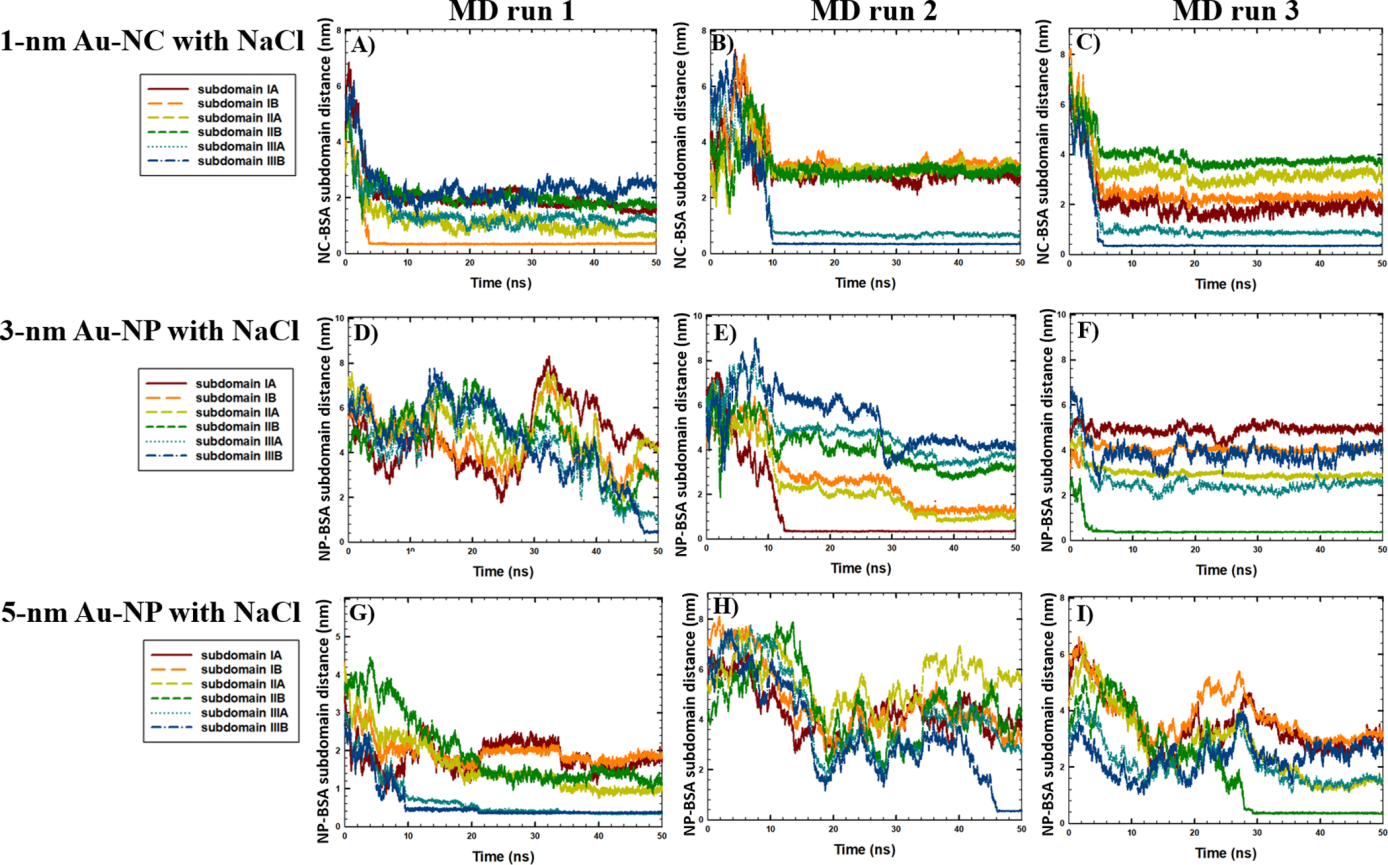
Time-evolution of the distances between the COM of the Au-NS and BSA monomer subdomains in the presence of 0.15 M NaCl in A) run 1 (1 nm Au-NC), B) run 2 (1 nm Au-NC), C) run 3 (1 nm Au-NC), D) run 1 (3 nm Au-NP), E) run 2 (3 nm Au-NP), F) run 3 (3 nm Au-NP), G) run 1 (5 nm Au-NP), H) run 2 (5 nm Au-NP), I) run 3 (5 nm Au-NP).

The binding sites of BSA were further studied considering six distinct subdomains of the BSA monomer to investigate the effect of Au-NS size on the binding mechanism associated with PC formation. The time-evolution of the intermolecular distances between the COM of the Au-NS and BSA subdomains in the absence and presence of NaCl is shown in Figures 5 and 6, respectively. According to the results, no specific binding sites for the BSA monomer were observed with Au-NPs of different sizes in the presence and absence of salt, as the 3 and 5 nm Au-NPs were bound to the various BSA subdomains in different runs. Our results are consistent with the MD study performed by Shao and Hall,^39^ who showed that 4 nm Au-NP binds to human serum albumin (HSA) in various regions. In addition, the results were in agreement with the experimental study of Dixon and Egusa,^40^ who reported multiple Au-binding sites in BSA based on the fluorescence spectroscopy of BSA-Au complexes.

**Figure 7:**
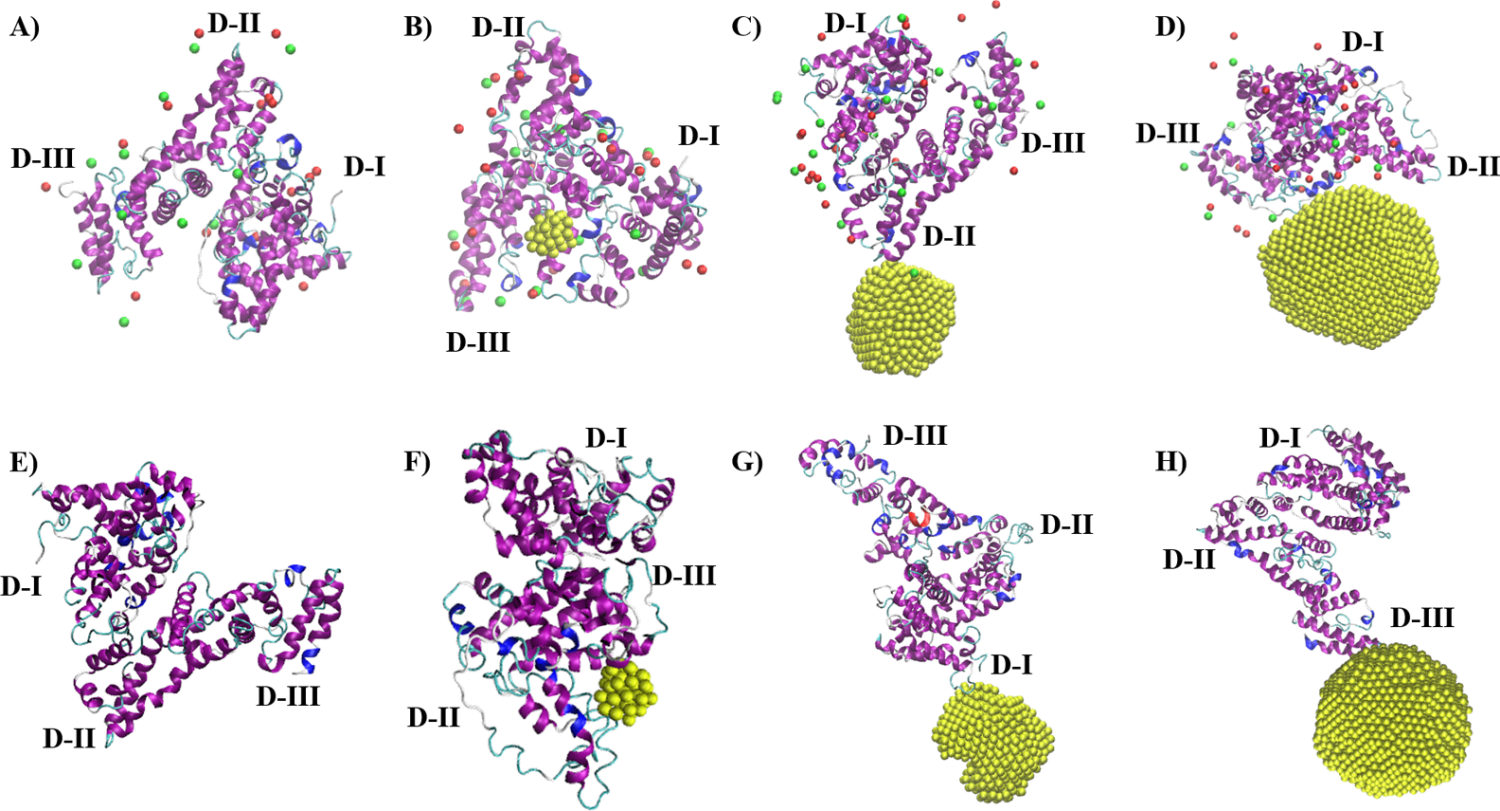
Representative snapshots of the simulated systems at the end of the simulations depicted from a single 50 ns run (yellow: Au; red: Na^+^ ions within 1 nm around the protein; green: Cl^–^ ions within 1 nm around the protein; secondary structure of the BSA protein: blue, red, violet: helices; yellow and cyan: unstructured bend and turn, D-I: Domain I, D-II: Domain II, D-III: Domain III; water molecules are not shown for clarity): (A) BSA monomer in the presence of NaCl, (B) BSA monomer and 1 nm Au-NC in the presence of NaCl, (C) BSA monomer and 3 nm Au-NP in the presence of NaCl, (D) BSA monomer and 5 nm Au-NP in the presence of NaCl, (E) BSA monomer in the absence of NaCl, (F) BSA monomer and 1 nm Au-NC in the absence of NaCl, (G) BSA monomer and 3 nm Au-NP in the absence of NaCl, (H) BSA monomer and 5 nm Au-NP in the absence of NaCl.

Furthermore, considering the effect of Au-NS with the smallest size, in the absence of NaCl (Figure 5A–C), the binding of Au-NC occurred with BSA subdomains IIA-B (Domain II) and subdomain IIIA (Domain III), associated with the intercalation of Au-NC inside the BSA monomer structure. This observation was also correlated with the significant loss of helical structures and decline in H-bonding in the presence of 1 nm Au-NC without NaCl (Table 1). In comparison, according to Figure 6A–C, the rapid aggregation of 1 nm Au-NC and BSA within the first 5–10 ns of the simulations was observed in the presence of NaCl, with the binding sites located at subdomain IB (Domain I) and subdomains IIIA-B (Domain III). Considering that no unfolding effect was observed in the presence of NaCl, a comparative stabilizing impact of salt on the BSA monomer was associated with the rapid binding of Au-NC to Domains I and III, with no significant intercalation into the protein structure. Representative snapshots of the simulated systems with Au-NS observed at the end of the 50 ns MD runs are shown in Figure 7.

## 3 Conclusions

In summary, the synergistic effect of Au-NP and NaCl on the secondary structure of BSA was investigated by using spectroscopic analysis and MD simulations. Although the CD spectroscopy showed BSA conformation stability in the presence of Au-NP without NaCl, a loss in the helical content was observed in the presence of Au-NP and NaCl. In addition, the ThT fluorescence assay showed that the Au-NPs suppressed the formation of the β-sheets in the BSA structure in PBS. Although β-sheets could not be observed in the BSA monomeric structure via MD simulations, the results showed induced changes in the BSA conformation in the presence of 5 nm Au-NP and NaCl, indicating the initial binding mechanism of a BSA monomer and a single Au-NP during the protein corona formation.

MD simulations further elucidated the size effect of Au-NP with diameters of 3 and 5 nm, and Au-NC with a size of 1 nm on their aggregation with the BSA monomer, considering the protein-binding sites and protein structural stability. Although the formation of stable BSA-NP conjugates was observed within 50 ns of the MD runs, no specific binding sites of the BSA monomer to Au-NPs of different sizes were revealed. In addition, due to the small size, 1 nm Au-NC destabilized the BSA monomer structure more significantly by binding to Domains II and III, decreasing the helical content in the BSA monomer, in the absence of NaCl. Interestingly, high aggregation kinetics of 1 nm Au-NC to BSA Domains I and III were observed in the presence of NaCl with no significant changes in the BSA conformation.

Overall, considering the growing importance of biocompatible tailored nanomaterials for various biomedical applications, the effects of NPs on protein structures and protein-NP interactions under physiological conditions are of great interest. Consequently, the results of our combined experimental and computational study provided insights into the synergistic effect of Au-NS and NaCl on the BSA structure, which might be further implicated in the biomedical applications of gold nanosized substances.

## 4 Methods

### 4.1 Materials

BSA, Au-NPs (diameter of 5 nm), and deuterium oxide (D_2_O) were purchased from Sigma-Aldrich (St Louis, MO, USA). Sodium chloride (NaCl) was purchased from Fujifilm Wako Pure Chemical Co. (Osaka, Japan) and ThT (Basic Yellow 1) was purchased from Tokyo Chemical Industry Co. (Tokyo, Japan). PBS was purchased from Gibco, Life Technologies Co. (Grand Island, NY, USA).

### 4.2 Circular Dichroism (CD) Spectroscopy

Thirty μg/mL BSA in H_2_O, 20 μg/mL Au-NP in PBS, and 90 mg/mL NaCl in H_2_O were used to perform CD spectroscopy measurements. The CD spectroscopy measurements were reported for four samples investigated in this study: BSA alone, BSA with NaCl, BSA with Au-NPs, and BSA with NaCl with Au-NP. The final concentration of BSA in the analyzed solutions was 15 μg/mL. The 5 nm Au-NP and NaCl concentrations were 1.33 μg/mL and 9 mg/mL, respectively. The samples were stirred and incubated for 24 h at 25 °C. A 3 mL sample was placed in a quartz glass cuvette with a path length of 10 mm (Q-4 Sansyo Co. Ltd., Tokyo, Japan). The measurements were performed with a circular dichroism spectrometer (J-820, JASCO CO., Tokyo, Japan).

### 4.3 ThT Fluorescent Measurement

Thirty μM BSA and 50 μM ThT were dissolved in PBS with or without 10 nM of Au-NPs (diameter = 5 nm). Each mixture was stirred for up to 2 h to analyze the rapid reaction of protein structure and Au-NPs. Each sample was irradiated with excitation light at 442 nm, and fluorescence was observed at 400–600 nm. The measurements were performed three times, and the average values were reported. It should be noted that although a plasmon absorption band at 510–560 nm was observed in 10 μM Au-NP, no light absorption was observed by Au-NP at lower concentrations (<1 μM). Considering that the concentration of Au-NP used in our ThT experiments is lower (10 nM Au-NP), the plasmonic effect of Au-NP would not affect the results of our experiments.

### 4.4 MD Simulations

To perform atomistic MD simulations, the Gromacs 2021.3 software^41^ was used with the Amber99SB forcefield, which was selected based on the validations from the literature.^29^ The protein structure of the BSA monomer was obtained from the Protein Data Bank (PDB ID: 4F5S)^42^ with a total charge of −16. The BSA structure has a high helical content and consists of 583 amino acids. The protein monomer is usually divided into three domains (Domain I: with the amino-acid residues number 1–196, Domain II: a. a. 204–381, and Domain III: a. a. 382–571), and six subdomains (IA: with the amino-acid residues number 1–107, IB: a. a. 108–196, IIA: a. a. 204–295, IIB: a. a. 296–381, IIIA: a. a. 382–493, IIIB: a. a. 494–571).

The system was neutralized by the addition of Na^+^ ions. The face-centered cubic structure of Au-NS was built by the simulation input generator CHARMM-GUI^43^ and the parameters for the gold atoms were taken from the literature: δ = 0.2951 nm and ε = 21.9006 kJ/mol.^44^ The simulations were performed in a 15 × 15 × 15 nm^3^ box with a randomly inserted BSA monomer and Au-NS. The average distance between the COM of the molecules, at the beginning of the simulations, was 3.8 nm. The simulation box was further solvated using TIP3P water molecules. To investigate the size effect of the Au-NS, simulations were performed with three distinct diameters: 1 nm (43 Au atoms), 3 nm (856 Au atoms), and 5 nm (3925 atoms), selected based on the limitations of the box size. The equimolar concentrations of the BSA monomer and Au-NPs were 0.5 mM. The mass concentration of the BSA monomer was 33 mg/mL, and the mass concentrations of the Au-NS were 4 mg/mL (*d*_NC_ = 1 nm), 84 mg/mL (*d*_NP_ = 3 nm), and 387 mg/mL (*d*_NP_ = 5 nm). To investigate the effect of the salt, simulations were performed in the absence and presence of 0.15 M NaCl. Representative snapshots of the initial configurations of the simulated systems with several molecules randomly inserted into the simulation boxes are shown in Figure S3 and Table S1.

The energy optimization step was performed by setting the maximum atomic force constraint at 100 kJ mol^–1^ nm^–1^. The constant-volume, constant-temperature (NVT) ensemble was conducted for 0.025 ns with hydrogen bonds (H-bonds) constraints at a constant temperature of 298 K, followed by a constant-pressure, constant-temperature (NPT) equilibration step performed for 0.025 ns with all-bond constraints at a constant pressure of 1 bar. For the pressure and temperature couplings, a Berendsen barostat and a V-rescale thermostat were applied, respectively,^45^ chosen based on previous literature studies.^29^ Periodic boundary conditions were applied for the XYZ directions. A cutoff of a 1 nm distance was used for the short-range interactions. The MD simulations were performed for 50 ns with an integration time step of 0.002 ps. The output parameters were saved for every 2000 frames, corresponding to 4 ns of dynamic runs. The systems with the BSA structure alone were simulated twice, whereas the systems with Au-NS were simulated three times starting from the random initial coordinates. Visual Molecular Dynamics (VMD) software^46^ was used to visualize the simulated systems.

The BSA monomer structure was characterized by SASA analysis. The secondary structure of the BSA monomer was analyzed using the “do_dssp” analysis (define the secondary structure of proteins), characterizing the average percentage amounts of coils, bends, turns, and helices observed in the last 25 ns of the simulations. The BSA binding sites were analyzed by studying the distances between the COM of the protein monomer and Au-NS. In addition, the average number of H-bonds (considering a bond angle of <30° and a bond distance of 0.35 nm) between the three different domains within the monomeric structure in the last 10 ns of the simulations was calculated. The SASA analysis was performed to characterize the possible folding or unfolding of the monomeric structure. The average values of different runs were reported for all of the mentioned types of analyses.

## Supporting Information Available

Representative snapshots of the initial structures at the beginning of the simulations, number of molecules in the simulated systems, and time evolution of total SASA, RoG, and intraprotein H-bonds of the BSA (PDF)
