# Supplementary material for: Effect of Gold Nanoparticles on the Conformation of Bovine Serum Albumin: Insights from CD Spectroscopic Analysis and Molecular Dynamics Simulations
Source: ACS Omega. 2024 Dec 3;9(50):49283–92. doi: 10.1021/acsomega.4c06409 (PMC11656231; doi:10.1021/acsomega.4c06409)
Supplement: Supplementary file 1 — ao4c06409_si_001.pdf [file ao4c06409_si_001.pdf]

Effect of gold nanoparticles on the conformation of bovine serum albumin: Insights from CD spectroscopic analysis and molecular dynamics simulations

Supporting Information

Samal Kaumbekova[1,2$], Naoya Sakaguchi[3$], Dhawal Shah[2], and Masakazu Umezawa[1,3*]

[1]Department of Medical and Robotic Engineering Design, Faculty of Advanced Engineering, Tokyo University of Science, 6-3-1 Niijuku, Katsushika, Tokyo 125-8585, Japan.

[2]Chemical and Materials Engineering, School of Engineering and Digital Sciences, Nazarbayev University, Kabanbay Batyr 53, Astana, 010000, Kazakhstan.

[3]Department of Materials Science and Technology, Graduate School of Advanced Engineering, Tokyo University of Science, 6-3-1 Niijuku, Katsushika, Tokyo 125-8585, Japan.

Key Words: gold nanoparticle; serum albumin; protein secondary structure; spectroscopic analysis; molecular dynamics simulations, protein corona

[$]These authors contributed equally

*Corresponding author: Masakazu Umezawa, Tokyo University of Science, 6-3-1 Niijuku, Katsushika, Tokyo 125-8585, Japan.

E-mail: masa-ume@rs.tus.ac.jp

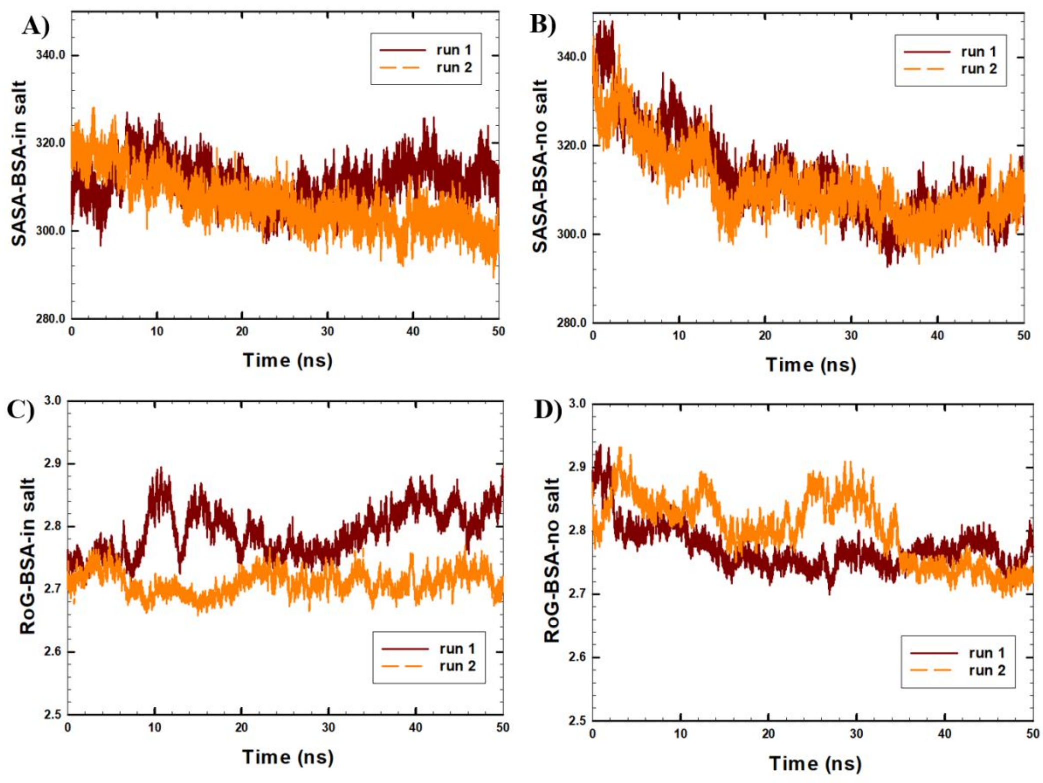

Figure S1. Time evolution of A) total SASA of the BSA monomer in the presence of 0.15 M NaCl, B) total SASA of the BSA monomer in the absence of salt, C) RoG of the BSA monomer in the presence of 0.15 M NaCl, D) RoG of the BSA monomer in the absence of salt.

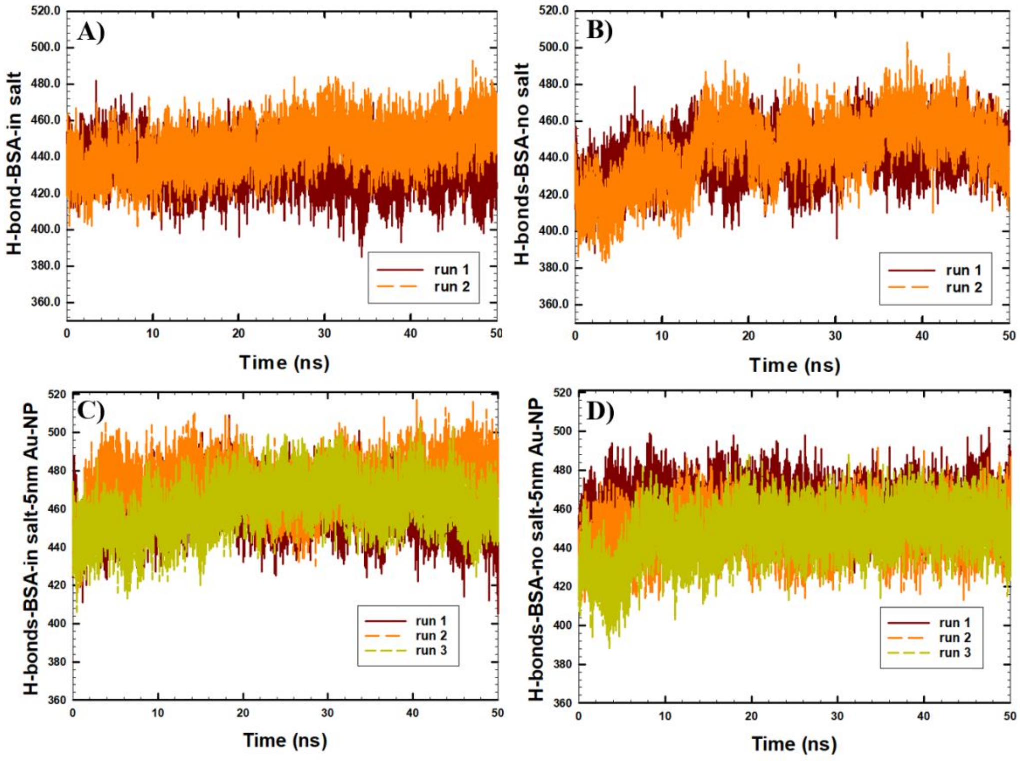

Figure S2. Time evolution of the intraprotein H-bonds A) in the system with no Au-NP, in the presence of 0.15 M NaCl, B) in the system with no Au-NP, in the absence of salt, C) in the system with 5-nm Au-NP, in the presence of 0.15 M NaCl, D) in the system with 5-nm Au-NP, in the absence of salt.

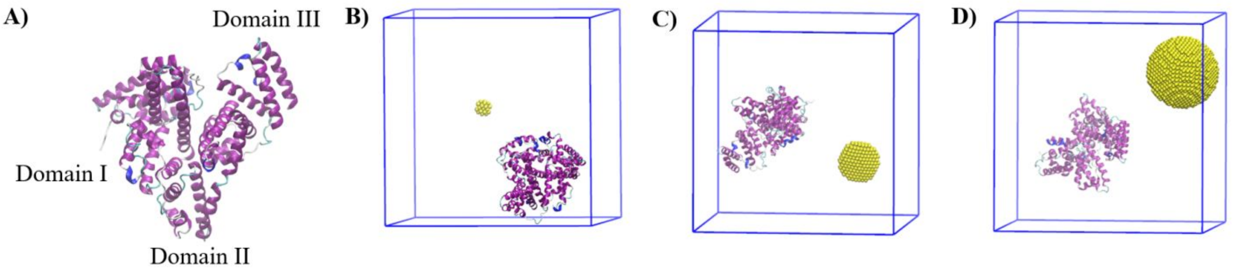

Figure S3. Representative snapshots of the initial structures at the beginning of the simulations (water and ions are not shown): A) BSA monomer, B) BSA monomer and 1-nm Au-NC, C) BSA monomer and 3-nm Au-NP, D) BSA monomer and 5-nm Au-NP.

Table S1. The average number of molecules in the simulated systems with BSA monomer in the absence and the presence of Au-NS: *in the presence of 0.15 M NaCl, **in the absence of salt.

*System	BSA	Au-NS	BSA: Au-NS mass ratio	H_2O	Na^+	Cl^-
BSA	1	0	-	105171	321	305
BSA + 1-nm Au-NC	1	1	1 : 0.12	105179	321	305
BSA + 3-nm Au-NP	1	1	1 : 2.55	104583	321	305
BSA + 5-nm Au-NP	1	1	1 : 11.73	102783	321	305

**System	BSA	Au-NS	BSA: Au-NS mass ratio	H_2O	Na^+	Cl^-
BSA	1	0	-	105754	16	-
BSA + 1-nm Au-NC	1	1	1 : 0.12	105771	16	-
BSA + 3-nm Au-NP	1	1	1 : 2.55	105235	16	-
BSA + 5-nm Au-NP	1	1	1 : 11.73	103433	16	-
